# Focal adhesion-related non-ciliary functions of CEP290

**DOI:** 10.1371/journal.pone.0325921

**Published:** 2025-07-09

**Authors:** Kazuhiko Matsuo, Yoshiro Nakajima, Masaki Shigeta, Daisuke Kobayashi, Shinichiro Sakaki, Satoshi Inoue, Naoki Takeshita, Atsuko Ueyama, Kousuke Nishikawa, Rie Saba, Hideya Yamasaki, Kei Yamada, Takahiko Yokoyama, Kenta Yashiro

**Affiliations:** 1 Department of Anatomy and Developmental Biology, Graduate School of Medical Science, Kyoto Prefectural University of Medicine, Kyoto, Japan; 2 Department of Pediatrics, Graduate School of Medicine, University of Tokyo, Hongo, Bunkyo-ku, Tokyo, Japan; 3 Department of Pediatrics, Graduate School of Medical Science, Kyoto Prefectural University of Medicine, Kyoto, Japan; 4 Department of Pediatrics, Graduate School of Medicine, Osaka University, Yamadaoka, Suita, Japan; 5 Department of Radiology, Graduate School of Medical Science, Kyoto Prefectural University of Medicine, Kyoto, Japan; Astellas, UNITED STATES OF AMERICA

## Abstract

Nearly all differentiated mammalian cells possess primary cilia on their surface. Ciliary dysfunction causes ciliopathy in humans. Centrosomal protein 290 (CEP290), a ciliary protein implicated in ciliopathies, localizes to the ciliary base and the centrosome in ciliated cells. CEP290-related ciliopathies arise from molecular dysfunctions of the CEP290 molecule, exhibiting a diverse range of symptoms. Thus far, these disorders have been attributed to cilia-specific functional abnormalities of CEP290, reflecting the conventional view of its primary role within cilia. However, CEP290 is also expressed in proliferating non-ciliated cells and localizes to the centrosome, suggesting potential cilia-independent functions of CEP290 in the pathophysiology of these disorders. In this study, we investigated the cilia-independent functions of CEP290 in non-ciliated cells. Our findings reveal that the loss of *Cep290* function impairs microtubule elongation due to malfunction of the microtubule organizing center. Notably, CEP290 forms a complex with adenomatous polyposis coli (APC), a protein that localizes to the centrosome and associates with microtubules. Importantly, reduced focal adhesion formation appears to underlie the phenotypic abnormalities observed in *Cep290* knockout cells, including impaired collective cell migration, altered cell morphology, and reduced adhesive capacity to the extracellular matrix. The APC-CEP290 complex plays a consistent and crucial role in stabilizing a focal adhesion molecule, paxillin, at the leading edge in non-ciliated cells. These findings provide a novel framework for understanding the molecular mechanisms underlying ciliopathies, highlighting the importance of CEP290’s cilia-independent functions.

## 1. Introduction

The centrosome is a non-membranous organelle that serves as a microtubule-organizing center (MTOC) and regulates cell polarity, intracellular trafficking, and migration via microtubules in interphase cells. During mitosis, the centrosome organizes the mitotic spindle microtubules for faithful chromosome segregation [1]. In differentiated cells or those in the G0 phase, the centrosome relocates near the plasma membrane, where it functions as a basal body, anchoring the formation of primary cilia. Primary cilia are vital for sensing and transmitting extracellular signals, thereby regulating diverse cellular processes. Functional abnormalities of primary cilia cause “ciliopathies,” a group of rare inherited human diseases typically affecting multiple organs [2]. Ciliopathy symptoms generally result from defective ciliary signaling and impaired cell motility. Impaired cell motility in ciliopathies has primarily been attributed to abnormal ciliary signaling. Primary cilia play a central role in transducing signals from pathways such as SHH, WNT, and PDGF, which regulate cell polarity, migration, and cytoskeletal dynamics [3]. Disrupted ciliary signaling can negatively affect actin organization and focal adhesion dynamics, thereby impairing cell migration.

Focal adhesions are specialized structures that mediate cell–ECM attachment and coordinate with the actin cytoskeleton to regulate cell motility [4]. Paxillin, a cytoskeletal protein, participates in cell adhesion and migration by functioning as a scaffold at focal adhesions, interacting with integrins, signaling molecules, and the actin cytoskeleton to maintain cell motility and stability [5,6]. Adenomatous Polyposis Coli (APC) plays a crucial role in regulating focal adhesion dynamics by mediating the transport and localization of paxillin to focal adhesions [7]. APC binds to microtubules and accumulates at their plus ends near the leading edge of migrating cells, where it associates with focal adhesions and promotes their assembly. This process involves APC’s interaction with microtubule plus-end tracking proteins and its ability to nucleate actin filaments, aiding the delivery and stabilization of paxillin at adhesion sites [8]. Disruption of APC function compromises focal adhesion turnover and cell migration, emphasizing its essential role in coordinating cytoskeletal dynamics and adhesion complex formation [9].

Centrosomal protein 290 (CEP290), a primary cilia protein, localizes to the transition zone at the base of primary cilia [10]. Mutations in this gene are responsible for ciliopathies, including nephronophthisis (NPHP) [11], Bardet–Biedl syndrome (BBS) [12], Joubert syndrome (JS) [10,13], Senior-Løken syndrome (SLS) [14], and Meckel syndrome (MKS) [15,16]. CEP290 is essential for initiating ciliary transition zone assembly [17], maintaining the molecular integrity of primary cilia [18], and forming aggresomes, where misfolded proteins, ubiquitin, molecular chaperones, and proteasomes are concentrated at the centrosome and the MTOC [19]. However, the phenotypic outcomes of *Cep290* mutations are highly variable, with patients presenting a diverse range of clinical features, such as polycystic kidney disease, retinitis pigmentosa, and skeletal dysplasia. This variability complicates understanding how a single gene mutation can cause various disease manifestations.

CEP290 also localizes to the centrosome in non-ciliated cells [13,20,21], suggesting that CEP290 likely has a cilia-independent function. Based on this evidence, we hypothesize that the diverse phenotypes resulting from CEP290 dysfunction are due to a combination of cilia-dependent and cilia-independent mechanisms, leading to a wide range of ciliopathic manifestations. In this study, we investigate the molecular function of CEP290 in non-ciliated cells. Our findings reveal that CEP290 localizes not only at the centrosome but also along microtubules. Both centrosomal and microtubular CEP290 regulate microtubule growth, which is essential for collective cell migration and adhesion. Importantly, the APC-CEP290 complex stabilizes paxillin, a key component of focal adhesions, at the focal adhesion plaques. These findings provide novel insights into the pathophysiology of ciliopathies by highlighting a cilia-independent role of CEP290.

## 2. Materials and methods

### 2.1. Establishment of *Cep290* knockout (KO) and PA-tagged CEP290 cell lines

Murine inner medullary collecting duct 3 (mIMCD3) cells (ATCC) were cultured in “growth conditions” with Dulbecco’s Modified Eagle’s Medium (DMEM)/Ham’s F12 (1:1) medium containing 10% fetal bovine serum, 100 U/mL penicillin G, and 100 µg/mL streptomycin, without inducing primary cilia formation.

Two single guide RNA (sgRNAs) were designed to establish the *Cep290*-null mutant cell lines using the CRISPR/Cas9 system [22]. The synthesized oligonucleotides are listed in Table 1.

**Table 1:** Oligonucleotides used to produce single guide RNA.

gRNA-CEP290-1S	5′-CACCTAGGTGGCTCAGCAGTCTGCAG-3′
gRNA-CEP290–1AS	5′-AAACCTGCAGACTGCTGAGCCACCTA-3′
gRNA-CEP290-2S	5′-CACCTAGGAAGCGCTTGTGCATCAGT-3′
gRNA-CEP290–2AS	5′-AAACACTGATGCACAAGCGCTTCCTA-3′

The oligonucleotides of the complementary sense (S) and antisense (AS) strands were annealed, and the 5′ end was phosphorylated with T4 polynucleotide kinase (Cat. #2021S; Takara, Kyoto, Japan). This phosphorylated DNA fragment was ligated to the *Bbs*I cloning site in pSpCas9(BB)-2A-Puro (PX459) V2.0 (Addgene plasmid #62988) [23], and the constructs corresponding to 1S/1AS and 2S/2AS were designated as pX459-sg*Cep290*-KO1 and pX459-sg*Cep290*-KO2, respectively. pX459-sg*Cep290*-KO1 or pX459-sg*Cep290*-KO2 was independently transfected into mIMCD3 cells using Lipofectamine 2000 (Thermo Fisher Scientific, Waltham, MA, USA) according to the manufacturer’s protocol. Puromycin-resistant clones were isolated using 2 µg/mL puromycin 48 hours after transfection. The genotype of the isolated clones was confirmed by sequencing PCR products amplified from the targeted genomic region. Table 2 lists the primers used for PCR and sequencing.

**Table 2:** List of primers used for puromycin-resistant clones PCR and sequencing.

CEP290-1F	5′-GCAGACTGAAAATTTACAGTGGAGTAC-3′
CEP290-1R	5′-CTCTATCTTTTTGTTCCAGCTGCTTC-3′
CEP290-2F	5′-ATGAGAAGATTGAAGTTCAGAACCAAG-3′
CEP290-2R	5′-CTAGTTTCAAACACACAGCAATGAATG-3′

A PA-tagged CEP290 cell line was established using a non-homologous end joining (NHEJ)-based method, as previously described with modifications [24] (see S1 Fig). Briefly, synthesized oligonucleotides were used to clone the sgRNA template into pX459, which was designated as pX459_sgRNA-CEP290-N_sgRNA-tia1l. The NHEJ knock-in donor plasmid was constructed and designated as pDonor-N-Hygro^R^-T2A-PA tag. These two plasmid constructs were co-transfected into mIMCD3 cells, and drug-resistant clones were isolated using 2 µg/mL puromycin and 25 µg/mL hygromycin D. The genotype of the isolated clones was confirmed by sequencing genomic PCR products. The primers used are listed in Table 3.

**Table 3:** Oligonucleotides used for CRISPR/Cas9 and genotyping.

Oligonucleotides for CRISPR/Cas9 mediated gene targeting	
BbsI_gRNA-tia1l_S	5′-CACCGGTATGTCGGGAACCTCTCC-3′
BbsI_gRNA-tia1l_AS	5′-AAACGGAGAGGTTCCCGACATACC-3′
gRNA-CEP290-N tag_S	5′-CACCGAGATGCCACCTAATATAAAG-3′
gRNA-CEP290-N tag_AS	5′-AAACCTTTATATTAGGTGGCATCTC-3′
**PCR/Sequencing primers for genotyping**	
CEP290-N tag-F	5′-GCCTGTGTCAGGGGAACTAA-3′
CEP290-N tag-R	5′-TCCACCTAAGGAAACAAACACA-3′

**Table 4:** List of antibodies used in this study.

Antibody	Cat. No.	Manufacturer	Dilution factors
Anti-CEP290 rabbit polyclonal antibody	NB100–86991	Novus Biologicals, Centennial, CO, USA	x500 (WB), x300 (ICC)
Anti-p150^Glued^ mouse monoclonal antibody	P41920	BD Bioscience, Franklin Lakes, NJ, USA	x1000 (WB), x500 (ICC)
Anti-α-tubulin mouse monoclonal antibody, clone DM1A	05-829	Merck, Darmstadt, German	x1000 (ICC)
Anti-α-tubulin rabbit polyclonal antibody	PM054	MBL, Tokyo, Japan	x500 (ICC)
Anti-γ-tubulin mouse monoclonal antibody, clone GTU88	T5326-200U	Merck	x300 (ICC)
Anti-paxillin mouse monoclonal antibody, clone 5H11	05-417	Merck	x500 (ICC)
Anti-PA rat monoclonal antibody	012-25863	Fujifilm, Tokyo, Japan	x1000 (WB), x500 (ICC)
Anti-APC rabbit polyclonal antibody	2504S	Cell Signaling Technology, Danvers, MA, USA	x1000 (WB), x500 (ICC)
Goat anti-rabbit IgG antibody-Alexa Fluo 488	A-11008	Thermo Fisher Scientific	x5000 (ICC)
Goat anti-mouse IgG antibody-Alexa Fluo 488	A-11001	Thermo Fisher Scientific	x5000 (ICC)
Goat anti-mouse IgG antibody-Alexa Fluo 555	A32727	Thermo Fisher Scientific	x5000 (ICC)
Goat anti-mouse IgG antibody-Alexa Fluo 633	A-21052	Thermo Fisher Scientific	x5000 (ICC)
Goat anti-rat IgG antibody-Alexa Fluo 555	A48263	Thermo Fisher Scientific	x5000 (ICC)

WB: Western blotting, ICC: Immunocytochemistry

### 2.2. Antibodies and immunostaining

The antibodies used in this study are listed in Table 4. Alexa Fluor 633 Phalloidin was used for F-actin staining (A22284; Thermo Fisher Scientific). Nuclei were stained with 4′,6-diamidino-2-phenylindole dihydrochloride (DAPI) (340–07971; DOJINDO, Kumamoto, Japan).

For immunostaining, cells were fixed with 4% paraformaldehyde (PFA) in phosphate-buffered saline (PBS) without divalent cations for 30 min at 25 °C or in methanol for 5 min at −20 °C. After removing the fixative agent by washing with PBS, the specimens were immersed in a blocking solution (PBS containing 5% normal goat serum, 0.03% TritonX-100, and 0.1% sodium azide) at 25 °C for 60 min. Subsequently, a primary antibody was applied to the specimens at an indicated dilution in the blocking solution. After incubation for 60 min at 25 °C, the redundant antibody was removed by washing with PBS-T (PBS containing 0.03% Triton X-100), a secondary antibody and DAPI, appropriately diluted in the blocking solution, were then applied to the specimens. After 30 min of incubation at 25 °C, the excess antibody and DAPI were removed by washing with PBS-T. The specimens were mounted using FluorSave reagent (345789; Merck). Images were acquired using an LSM900-Airyscan confocal laser scanning microscope (ZEISS, Oberkochen, Germany). Super-resolution images were acquired by Airyscan mode of this microscope. Line profiles and colocalization analysis were processed using the Zen software (ZEISS).

### 2.3. Microtubule regrowth assay

The microtubule regrowth assay was performed as previously described [25]. Briefly, wild-type, *Cep290* KO1, and *Cep290* KO2 cells were cultured on glass coverslips overnight (O/N) and then incubated on ice for 30 min to depolymerize the microtubules. Next, the cold culture medium was replaced with medium prewarmed to 37 °C. The cells were immediately transferred to a CO_2_ incubator at 37 °C and incubated for 3 min. The cells were subsequently fixed with cold methanol (−20 °C), followed by staining with anti-α-tubulin antibody (DM1A; #05–829, Merck). The specimens were observed under an IX83 fluorescence microscope (EVIDENT, Tokyo, Japan). The acquired images were processed using the cellSens Dimensions (EVIDENT) and ImageJ/Fiji software [26]. Fluorescence intensity at a defined distance from the centrosome was measured by cellSens Dimensions imaging software. Data from three independent experiments were statistically analyzed.

### 2.4. Cell migration assay

Wild-type and *Cep290* KO cells were treated with mitomycin C at a final concentration of 1 µg/mL to inhibit proliferation. A total of 5 × 10^4^ cells from each cell line were individually seeded into a Culture-Insert 2 well (ib80209, ibidi GmbH, Bayern, Germany) placed in a well of a 24-well plate (3820−024, Iwaki, Tokyo, Japan), creating a cell-free gap of 500 μm. Culture inserts were removed 24 h after seeding, and cell migration was observed using an In Cell Analyzer 2200 (Cytiva) every 6h over a 24-hour period. At each time point, the shortest distances across ten randomly selected gaps created by spacer removal were measured for wild-type and *Cep290* KO cells. Data were obtained from three independent experiments and statistically analyzed.

### 2.5. Cell attachment assay and nocodazole treatment

For specimen preparation, 1 × 10^5^ cells were seeded in a 6-well plate (140675, Thermo Fisher Scientific) containing glass coverslips coated with collagen IV (Nitta Gelatin, Inc., Osaka, Japan). For the cell attachment assay, the culture medium was replaced with PBS, and weakly adherent cells were removed by washing with PBS after 4h of incubation. The remaining cells on the glass coverslips were fixed with 4% PFA in PBS, followed by fluorescent immunocytological staining. For nocodazole treatment, nocodazole was added to the growth medium at a final concentration of 0.1 ng/mL, 24 h after seeding onto collagen IV-coated coverslips. The cells were incubated at 37 °C for 3 min, after which weakly adherent cells were removed by washing with PBS. The remaining cells were subsequently fixed with 4% PFA. Fixed cells were subjected to immunostaining. Images were acquired using APEXVIEW APX100 (EVIDENT). Cell and paxillin areas were measured using ImageJ/Fiji software [26]. Data were obtained from three independent experiments and statistically analyzed.

### 2.6. 3D live imaging of EB3-EGFP

Full-length cDNA of human EB3 was amplified by RT-PCR from total RNA harvested from hTERT-RPE1 (retinal pigment epithelial) cells, and the PCR products were cloned into the *Eco*RI/*Sal*I multiple cloning site of the pEGFP-N3 vector (Takara), designated as pEGFP-hEB3. pEGFP-hEB3 was transfected with the TransIT-LT1 reagent (Takara) into wild-type or *Cep290*-KO cells seeded in a glass-bottomed dish (Matsunami Glass Ind.). Live imaging data were acquired as Z-stack images using Zeiss LSM900-Airyscan confocal fluorescence microscope, as follows: Z = 0.336 µm × 5 slices (total 1.68 µm), total duration = 10 min (600 s) equivalent to 207 frames; frame time = 409.60 ms. The velocity of the EB3-EGFP positive foci was measured using Imaris software (Oxford Instruments) as follows. First, the acquired Z-stack 3D image files were processed with a Gaussian filter to reduce background noise. Following noise reduction, a spot model of EB3-EGFP was generated and analyzed for velocity using the Spots object tool in Imaris. Data were obtained from three independent experiments and statistically analyzed. The hTERT-RPE1 cell line was obtained from ATCC (CRL-4000).

### 2.7. Proximity ligation assay

The proximity ligation assay (PLA) was performed using the Duolink In Situ Orange Starter Kit Mouse/Rabbit (DUO92103; Sigma-Aldrich), according to the manufacturer’s protocol. Briefly, the cell specimens fixed with 4% PFA were treated with Duolink blocking solution for 60 min at 37 °C. Next, an anti-APC antibody (rabbit), appropriately diluted in the Can Get Signal A solution (TOYOBO, Osaka, Japan), was applied to the specimens. The specimens were washed twice with wash buffer A after incubation for 60 min at 25 °C. Anti-PA tag-PLUS PLA probe and anti-rabbit IgG-MINUS PLA probes (DUO92005; Sigma-Aldrich) appropriately diluted in the Duolink antibody diluent, were applied to the specimens which were then incubated for 60 min at 37 °C. Anti-PA-tag antibody conjugated with a PLUS oligonucleotide was prepared using the Duolink In Situ Probemaker PLUS (DUO92009; Sigma-Aldrich). The specimens were washed twice with wash buffer A and subsequently incubated with Duolink ligation solution for 30 min at 37 °C. After the ligation reaction, the specimens were washed again with wash buffer A and immersed in an amplification buffer containing polymerase. The amplification reaction was carried out in a pre-heated humidity chamber for 100 min at 37 °C. The specimens were then washed with wash buffer B and mounted for observation. Data were acquired using an IX83 immunofluorescence microscope (EVIDENT), and the obtained images were processed using cellSens Dimension software (EVIDENT).

### 2.8. Data analysis

Biologically triplicated data were statistically analyzed. Significant differences among multiple groups were analyzed via ANOVA with post hoc Tukey tests. The results are presented as means ± standard deviation (SD), along with *p* value. Statistical significance was defined as at *p* < 0.01 or 0.05. Data shown in Fig 4F and 4I were statistically analyzed by Student’s *t*-*t*est based on three independent experiments.

**Fig 1:**
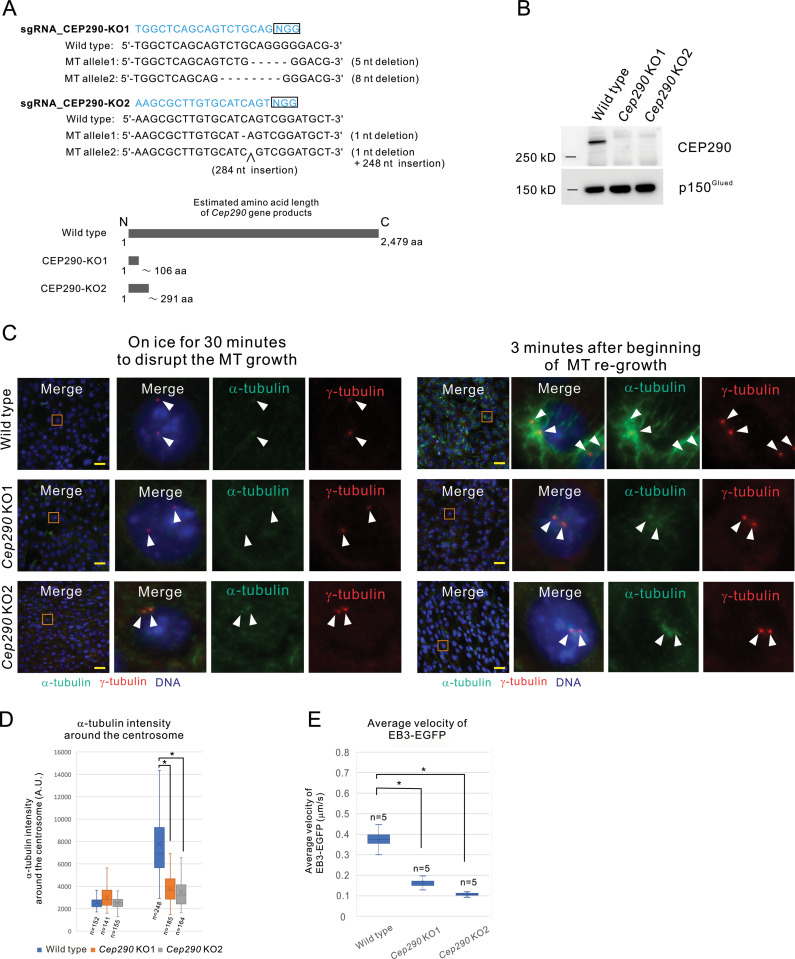
*Cep290* KO impairs the microtubule organizing center (MTOC) function of the centrosome. **A.** *Cep290* KO1 and *Cep290* KO2 allele. The boxed sequences represent the protospacer adjacent motif (PAM). The lower panel shows the expected amino acid length of the products from the KO alleles. **B.** The production of CEP290 in wild-type and *Cep290* KO cells was analyzed by western blotting. **C.** Microtubule regrowth assay. Incubation of the wild-type and *Cep290* KO cells on ice induced depolymerization of almost all microtubules, and α-tubulin was observed only at the centrosome. After restoring the temperature to 37 °C, microtubules were re-grown from the centrosome in wild-type cells, whereas significant regrowth of microtubules was not observed in *Cep290* KO cells. Green: α-tubulin, Red: γ-tubulin, and Blue: DNA. Representative cells, outlined with orange squares, are shown in magnified view on the right. Arrowheads indicate the centrosome. Scale bar indicates 40 μm. **D.** Fluorescence intensities of the α-tubulin around the centrosome in (C) were quantified. Data from three independent experiments were statistically analyzed. *: *p* < 0.01. **E.** Box plot represents EB1-EGFP velocity in the wild-type or *Cep290* KO cells. Data from three independent experiments were statistically analyzed. *: *p* < 0.01.

**Fig 2:**
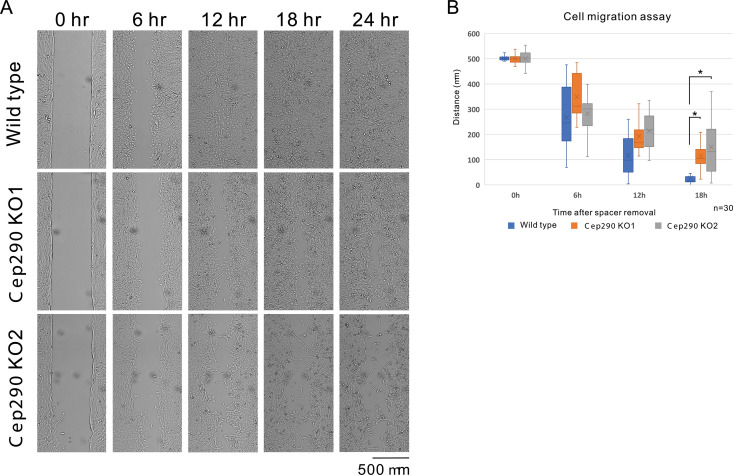
*Cep290* KO impairs collective cell migration. **A.** Data from cell migration assay are shown. Mitomycin C-treated cells were seeded, creating an initial gap of 500 μm in width, and monitored for up to 24 **h.** Notably, gaps in the *Cep290* KO cells took more time to close. The scale bar indicates 500 μm. **B.** The average gap distance was measured and statistically analyzed. The data were acquired from three independent experiments (n = 30 at each plot).

**Fig 3:**
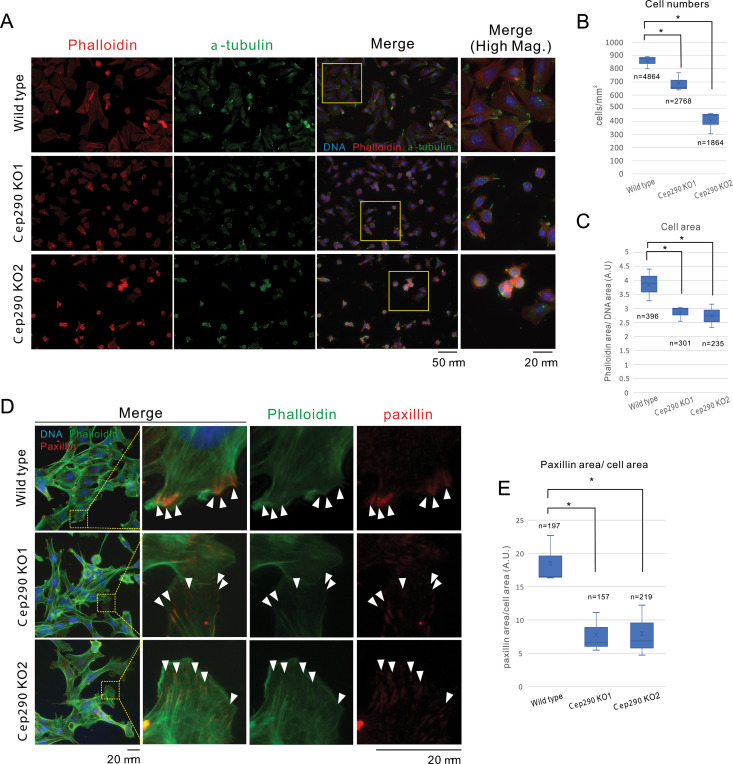
Reduced adhesive capacity in *Cep290* KO cells. **A.** *Cep290* KO cells exhibited reduced cell adhesion and appeared shrunken. The cells of the determined number were seeded on the glass coverslips coated with collagen IV, and those that failed to sufficiently adhere were removed 4 h after seeding. Cell number and morphology were observed. Magnified images, indicated by yellow squares, are displayed on the far right. Red: F-actin, Green: α-tubulin, and Blue: DNA. **B.** Quantification of cell numbers from the same experiment as in A revealed reduced adhesion in *Cep290* KO cells. Data represent three independent experiments. *: *p* < 0.01. **C.** *Cep290* KO cells exhibited shrunken morphology as seen in **A.** The graph shows the area occupied by cells, normalized to the area occupied by the nucleus. Data were analyzed in triplicate independent experiments. *: *p* < 0.01. **D.** Focal adhesion formation was diminished in *Cep290* KO cells. Green: F-actin, red: paxillin, and blue: nucleus. Scale bar indicates 20 μm. The boxed areas are magnified on the right. Arrowheads indicate focal adhesions. **E.** Quantification of the area occupied by paxillin, normalized to cell area in D was measured and analyzed in triplicate independent experiments. *: *p* < 0.01. Note that the focal adhesion was not significantly reduced in the KO (see S3 Fig).

## 3. Results

### 3.1. CEP290 is essential for the MTOC function of the centrosome

To elucidate the molecular functions of CEP290 in non-ciliated cells, we generated *Cep290* KO cell lines using the CRISPR-Cas9 system in mIMCD3 cells. To avoid misidentifying an off-target effect as specific phenotype, we generated two independent *Cep290* null alleles, *Cep290* KO1 and *Cep290* KO2, using differently designed sgRNAs (Fig 1A). CEP290 protein production was completely absent in both *Cep290* KO1 and KO2 cells (Fig 1B).

In non-ciliated cells, CEP290 localizes to the centrosome [13,20,21]. Because the centrosome is a major MTOC in mammalian cells, we examined whether CEP290 is required for MTOC function using a microtubule regrowth assay. To distinguish the cilia-independent role of CEP290, proliferative non-ciliated cells were deliberately used throughout the study (S1 Fig). In wild-type cells, microtubules were efficiently reassembled throughout the cytoplasm within 3 minutes of returning to regrowth conditions. In contrast, microtubules remained disrupted in all *Cep290* KO cell lines, with tubulin protein concentrated around the centrosome (Fig 1C and 1D). To further investigate microtubule dynamics in *Cep290* KOs, we measured the velocity of microtubule plus-end growth using EB3-EGFP, a member of the microtubule plus end-tracking proteins (+TIPs), tagged with GFP, was transiently expressed [27]. The velocity of EB3-EGFP movement reflects microtubule growth dynamics. Our analysis revealed that the velocity of EB3-EGFP was significantly slower in the *Cep290* KO cells than in wild-type cells (Fig 1E and S11–S13 Movies). Thus, CEP290 is essential for MTOC function and the proper growth of microtubules.

### 3.2. Malfunction of CEP290 affects collective cell migration

Proper regulation of microtubule dynamics is critical for collective cell migration, as MTOC malfunction disrupts normal microtubule dynamics. To assess whether collective cell migration of *Cep290* KO cells was impaired, we performed a cell migration assay. We monitored the closure of a 500-μm-wide gap in a cell monolayer composed of cells that had lost proliferative potential but remained non-ciliated due to treatment with mitomycin C (S2 Fig). Compared to wild-type cells, *Cep290* KO cells required significantly more time to fill the gap (Fig 2A and 2B).

Thus, as expected, CEP290 plays a significant role in collective cell migration, likely through its influence on microtubule dynamics by modulating MTOC function.

### 3.3. Cell adhesion capacity depends on CEP290

Microtubules are considered important for cell migration. However, the underlying molecular mechanisms remain unclear. Generally, microtubules are involved in cell migration by modulating actomyosin dynamics, transporting the required membrane components to the leading edge, and/or supplying molecules related to cell adhesion to adhesion plaques [28]. In our experiments, we noticed that the *Cep290* KO cells detached easily from the culture dish surface. This evidence suggests that CEP290 regulates cell adhesion to the extracellular matrix (ECM). To test this hypothesis, we assessed the adhesion of *Cep290* KO cells to type IV collagen, a component of the basement membrane, in comparison with wild-type cells. We seeded a defined number of cells on the glass surface coated with type IV collagen, incubated them for 4 h, and then washed to remove weakly adherent cells. Finally, we quantified the number of the cells that remained attached to the surface (Fig 3).

Consequently, *Cep290* KO cells exhibited reduced adhesion to the type IV collagen-coated surface compared to wild-type cells. Additionally, the *Cep290* KO cells appeared shrunken and smaller than wild-type cells (Fig 3A-3C). To further explore this aspect, we examined the status of the cell adhesion plaques, marked by paxillin.

Immunofluorescence micrographs revealed that the fluorescence intensity of paxillin, a key component of focal adhesions, was reduced in *Cep290* KO cells, particularly at the leading edge (Fig 3D and 3E). However, the number of paxillin foci per cell did not differ significantly between genotypes (S3 Fig). These results suggest that CEP290 may contribute to stabilizing focal adhesion dynamics.

### 3.4. CEP290 localizes to the leading edge and interacts with adenomatous polyposis coli (APC)

**Fig 4:**
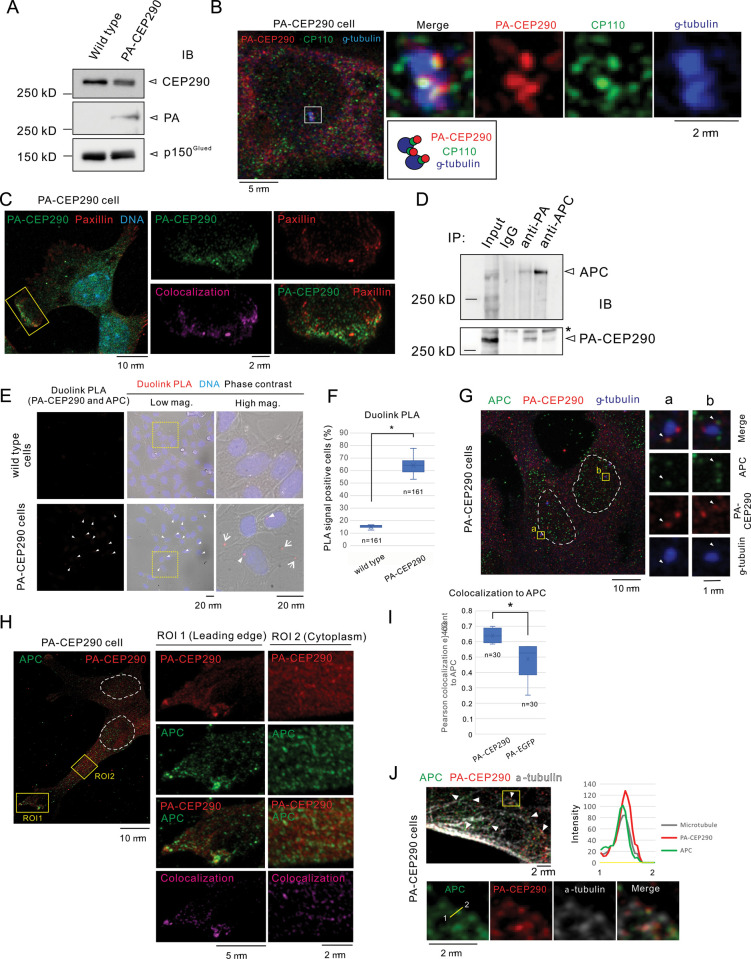
CEP290 forms a complex with APC on the microtubule network. **A.** PA-CEP290 cells, generated via CRISPR/Cas9-mediated insertion of a PA tag into the CEP290 locus, were validated by Western blot using anti-PA antibody, with p150^Glued^ as a loading control. **B.** Super-resolution microscopy shows PA-CEP290 colocalized with the centriole marker CP110 at the centrosome. Magnified boxed regions and corresponding pseudo-color images are shown. Schematics illustrations of centrosome structures are provided below. **C.** At the leading edge, PA-CEP290 was observed to colocalize with paxillin using super-resolution microscope. Magnified boxed regions are shown on the right side as indicated pseudo-colors. Colocalization area was analyzed by Zen software and shown as magenta. **D.** Co-immunoprecipitation confirmed reciprocal interactions between PA-CEP290 and APC. An asterisk indicates a non-specific band. **E.** Proximity ligation assay (PLA) demonstrates colocalization of PA-CEP290 and APC, visualized as red fluorescent signals. Centrosomal (arrowheads) and cytoplasmic (arrows) signals are shown with magnified views. **F.** Percentage of the PLA signal-positive cells relative to the total cells were quantified and analyzed (Student’s *t*-test, *p* < 0.01, n = 161). The data were obtained from three independent experiments. **G** Super-resolution microscopies show subcellular localization of APC (green), PA-CEP290 (Red) and γ-tubulin (blue). Centrosomes regions were magnified as boxed region **a** and **b**. APC colocalized with PA-CEP290 as indicated by arrowheads. The dashed lines indicate the nucleus. **H.** Super-resolution microscopies of magnified boxed regions at the leading edge (ROI1) and cytoplasm (ROI2). PA-CEP290 (red) partially colocalized with APC (green). Colocalization area was analyzed and shown as magenta. The dashed lines indicate the nucleus. **I.** Pearson’s colocalization coefficients for PA-EGFP/PA-CEP290 with APC were analyzed using Coloc2 in ImageJ (Student’s *t*-*t*est, *p* < 0.01, n = 30). The data were obtained from three independent experiments. **J.** Super-resolution microscopy of APC (green), PA-CEP290 (red), and α-tubulin (grey) shows coinciding fluorescence intensity peaks along the indicated line.

To further explore the relationship between CEP290 and paxillin, we tested whether CEP290 directly binds to paxillin. Unfortunately, we could not find any commercially available anti-CEP290 antibodies suitable for immunostaining and immunoprecipitation (IP) of specimens from mIMCD3 cells. As an alternative, we genetically modified the *Cep290* gene in mIMCD3 cells using the CRISPR-Cas9 system so that the N-terminus of CEP290 contains a PA tag (PA-CEP290) (S4–S5 Figs) [29]. We validated PA-CEP290 production by Western blotting (Fig 4A) and its subcellular localization by immunostaining (Fig 4B and 4C, and S4A–S4G Fig).

As expected, we found that PA-CEP290 was endogenously produced and localized to the centrosome, consistent with the previous findings [13,21] (Fig 4B). The centrosome is primarily composed of two centrioles, surrounded by pericentriolar material (PCM). Detailed analysis revealed that PA-CEP290 colocalized with CP110, a known centriole-associated protein, suggesting that CEP290 localizes to the centrioles [30] (Fig 4B). Furthermore, we did not observe any abnormalities in the morphology, growth or migration of the PA-CEP290 mIMCD3 cell line (S4H and S4I Fig). Thus, we conclude that PA-CEP290 functions physiologically similarly to endogenous CEP290. Using this cell line, we performed immunostaining and co-immunoprecipitation assays with anti-PA and anti-paxillin antibodies. Although PA-CEP290 colocalized with paxillin at the leading edge (Fig 4C), co-immunoprecipitation between PA-CEP290 and paxillin was not detected by Western blotting. These findings suggest that CEP290 does not directly bind to paxillin.

In contrast, a previous study showed that APC, a microtubule-associated protein and regulator of cell adhesion, is essential for paxillin localization at the leading edge [31]. Based on this, we hypothesized that CEP290 forms a complex with APC that facilitates the transport of paxillin to focal adhesions and/or stabilizes paxillin-based focal adhesions at the leading edge. To test this, we first assayed the interaction between CEP290 and APC by co-immunoprecipitation. Our results demonstrated that PA-CEP290 and APC were reciprocally co-immunoprecipitated, suggesting that CEP290 forms a complex with APC (Fig 4D).

To further investigate the interaction between APC and CEP290, we assessed whether these molecules closely colocalize within cells. Using the proximity ligation assay (PLA), which detects and visualizes interactions only when two target molecules are in close proximity, we found that CEP290 and APC interact at the centrosome and in the cytoplasm (Fig 4E and 4F). Importantly, super-resolution microscopy uncovered colocalization of CEP290 and APC sparsely throughout the cytoplasm, at the centriole, and at the leading edge (Fig 4G-4I), although many APC molecules not associated with CEP290 were also found. Furthermore, although there was no significant difference in the overall protein levels of APC between wild-type and *Cep290* KO cells, its localization at the leading edge was reduced in *Cep290* KO cells (S6 Fig). These findings indicate that APC and CEP290 indeed form a complex, but only a subset of APC molecules interacts with CEP290. Notably, overlapping fluorescent signals of APC, PA-CEP290, and α-tubulin suggest that APC-CEP290 complexes are associated with microtubules. This is supported by the evidence that the fluorescence intensity peaks of APC, CEP290, and α-tubulin were spatially aligned in line profile analysis (Fig 4J and S7 Fig). This evidence suggests that the APC-CEP290 complexes may be transported along the microtubule network. Given that APC binds to paxillin and is critical for its distribution [31], it is likely that CEP290 regulates paxillin transport via its interaction with APC.

### 3.5. Inhibition of focal adhesion turnover by nocodazole treatment did not restore the reduction of adhesion and shrunken morphology of *Cep290* KO cells

**Fig 5:**
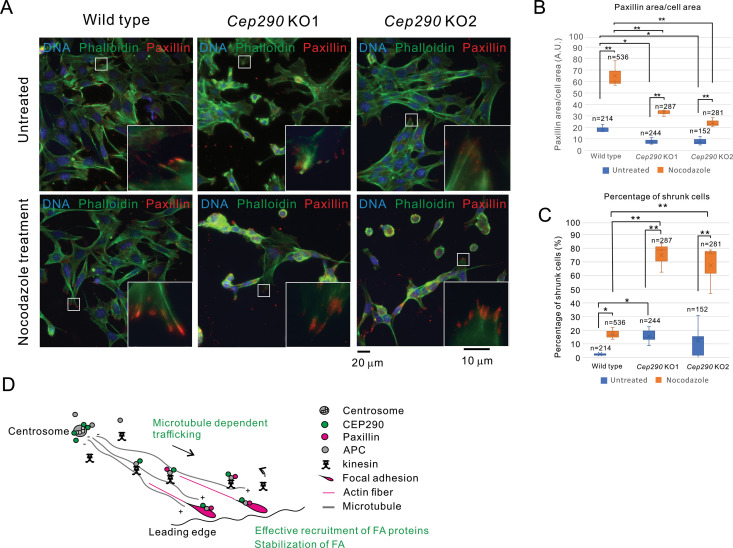
CEP290 regulates paxillin transport to focal adhesion/leading edge via supporting microtubule growth and forming a complex with APC. **A.** Adhesive capacity after nocodazole treatment was assessed using the same assay as in Fig 3A. Note that nocodazole treatment did not rescue the reduced adhesive feature of the *Cep290* KO. Red: F-actin, green: microtubule, and blue: nuclei. **B.** Nocodazole treatment failed to restore focal adhesion formation in *Cep290* KO cells. The percentage of shrunken cells remained unchanged (left panel), and the focal adhesion area marked by paxillin remained significantly smaller in *Cep290* KO cells compared to wild-type cells (right panel). Data represent biological triplicates. *: *p* < 0.05, **: *p* < 0.01. **C.** The graph shows the ratio of paxillin intensity with or without nocodazole treatment. Paxillin signal increased across all cell types following nocodazole treatment, indicating that nocodazole inhibited the turnover of paxillin as expected. Data represent biological triplicates. *: *p* < 0.05, **: *p* < 0.01. **D.** A schematic model illustrating the role of CEP290 in regulating focal adhesion dynamics (refer to the main text).

Nocodazole, a compound that binds to β-tubulin and disrupts microtubule assembly/disassembly dynamics, is known to enhance the stabilization and area expansion of focal adhesions by suppressing microtubule-dependent turnover of focal adhesion-related molecules [32]. To investigate whether nocodazole treatment could rescue the reduced adhesion capacity and impaired focal adhesion formation observed in the *Cep290* KO cells, we conducted further analyses (Fig 5A-5C). Notably, nocodazole treatment increased the intensity of intracellular paxillin signals in both wild-type and the *Cep290* KO cells (Fig 5B).

This observation, an increase in intracellular paxillin signal consistent with a previous report, supports that nocodazole functioned appropriately as expected. However, key phenotypes of *Cep290* KO cells, including shrunken cell morphology, reduced size and number of focal adhesions, were not rescued (Fig 5A–5C and S8A and S8B Fig). These findings suggest that paxillin accumulation at individual adhesion plaques is insufficient to support effective cell adhesion when the overall size and number of focal adhesions are diminished.

## 4. Discussion

Primary cilia play a critical role in transmitting extracellular signals into the cell. Ciliary protein dysfunction may cause ciliopathies, which are a group of disorders characterized by diverse phenotypes. Although some phenotypes are commonly observed across different ciliopathies, others are specific to individual conditions.

In this study, we hypothesized that certain phenotypes of ciliopathies directly result from primary cilia dysfunction, whereas others occur through indirect mechanisms. CEP290 is a key ciliary protein involved in the regulation of ciliary signaling. Mutations in *Cep290*, a gene related to ciliopathy, result in a wide range of phenotypes, possibly due to the complex interplay between primary ciliary dysfunction and other cellular defects [33]. To investigate this, we analyzed CEP290 function in non-ciliated cells, independent of its role in primary cilia.

Our findings show that CEP290 is required for regulating MTOC, collective cell migration, and cell adhesion to ECM (Fig 5D). Notably, CEP290-dependent focal adhesion formation appears vital for both collective cell migration and ECM adhesion. CEP290 forms a complex with APC and likely facilitates the transport of paxillin molecules toward focal adhesions and the leading edge by binding to APC and/or stabilizing paxillin-based focal adhesions. Previous studies have shown that APC contributes to microtubule stability [34]. It is possible that suppression of CEP290 expression impairs APC function, resulting in microtubule instability and disrupting the transport of focal adhesion molecules. Furthermore, a previous study indicates that CEP290 directly interacts with microtubules through its myosin tail homology domain and contributes to microtubule stabilization [35]. Therefore, the phenotypes observed in *Cep290* KO cells may be partly attributed to microtubule instability. In addition, it is conceivable that CEP290, similar to its role in microtubule stabilization, may also stabilize focal adhesions through an unknown mechanism. These findings strongly suggest that processes such as collective cell migration, microtubule growth, and cell adhesion contribute to certain CEP290-related ciliopathy phenotypes. Importantly, cell migration defects resulting from malfunction of ciliopathy-related molecules have generally been considered a consequence of impaired ability to sense signals, such as SHH, WNT, or PDGF, via primary cilia [36–38]. Therefore, our findings of a cilia-independent migration defect represent a novel perspective on the molecular pathophysiology of ciliopathies.

Given that APC is involved in WNT signaling, our findings suggest that CEP290 may also play a role in WNT signaling. In non-ciliated cells, canonical WNT signaling stabilizes cytoplasmic β-catenin, leading to increased nuclear β-catenin levels. Nuclear β-catenin initiates the transcription of TCF/LEF1-responsive genes. In this context, if WNT signaling is inactive, the Axin-GSK3β-APC destruction complex interacts with β-catenin and promotes its degradation, thereby suppressing the WNT signaling pathway. Although we did not clarify whether CEP290 is involved in WNT signaling via APC, we cannot exclude this possibility. Further studies are required to address this issue.

Our study showed that the effective trafficking of paxillin to focal adhesions requires a molecule that seems to play an important role at the centrosome and cilium. Previous studies have revealed that (1) paxillin [39] and (2) APC localize to the centrosome, contribute to microtubule growth, and directly interact with both paxillin and focal adhesion kinase [31,40–43]. Therefore, it is reasonable to conclude that the centrosome protein CEP290 is directly involved in the regulation of paxillin. Interestingly, nocodazole treatment, which suppresses paxillin turnover, did not restore the impaired cell adhesion capacity of *Cep290* KOs (Fig 5A-5C). Despite the presence of abundant paxillin molecules at focal adhesions, effective cell adhesion to the ECM cannot be maintained if the size and number of focal adhesions are reduced, as observed in *Cep290* KO cells.

Although issues related to primary cilia have been extensively studied in the context of ciliopathy pathogenesis, our understanding of the molecular pathogenesis of ciliopathies remains incomplete. Nevertheless, our study provides a novel platform to deepen our knowledge of the function of non-primary cilia in ciliopathy-related molecules. Further analyses are needed to clarify the roles of primary ciliary proteins in primary cilia and in non-ciliary contexts to better understand the molecular pathogenesis of ciliopathies.

## Supporting information

S1 Fig Culture condition of mIMCD3. In this study, we used non-ciliated mIMCD3 cells. This cell line can form primary cilia under serum starvation in confluent condition. On the other hand, in normal culture medium, containing fetal bovine serum and under sparse condition, almost all cells do not form primary cilia. (A) Immunostaining of non-ciliated (left) and ciliated (right) mIMCD3 cells. The scale bar represents 20 μm. Green (Arl13b; cilia marker), Red (γ-tubulin; centrosome) and Blue (DAPI; nucleus). (B) The percentage of the ciliated cell among the cells cultured in growth medium was quantified, compared to those under serum starvation conditions, and statistically analyzed using Student’s *t*-test. Experiments were performed independently in triplicate. *: *p* < 0.01.
(EPS)

S2 Fig Mitomycin C-induced non-proliferative conditions do not promote ciliogenesis. Related to Fig 2, we evaluated the percentage of non-ciliated cells following mitomycin C treatment. Cells were subjected to the same experimental procedure as in Fig 2, then fixed with 4% PFA in PBS and processed for immunostaining. (A) Immunofluorescence images show staining with anti-Arl13b (green), anti-γ-tubulin (red), and DAPI (blue). The scale bar represents 20μm. After mitomycin C treatment, more than 90 percent of both wild-type and *Cep290* KO cells lacked cilia, similar to untreated controls. Rare, short cilia observed in a small number of cells are indicated by white arrowheads. (B) Quantification of the percentage of non-ciliated cells in wild-type and *Cep290* KO cells, with or without mitomycin C treatment. Statistical analysis was performed using ANOVA with post-hoc Tukey tests. Data represent the mean ± SD from three independent experiments. N.S. indicates no significant difference.
(EPS)

S3 Fig Functionally null mutations in *Cep290* do not alter the number of paxillin foci per cell. Related to Fig 3D, we analyzed the number of focal adhesions per cell of wild-type and *Cep290* KO cells. Data were obtained from three independent experiments and quantified by ImageJ/Fiji. Statistical significance among multiple groups was assessed via ANOVA with post-hoc Tukey tests. N.S. indicates no significant difference.
(EPS)

S4 Fig The strategy of tagging CEP290 with PA tag. (A) We designed sgRNA-CEP290-N and sgRNA-tia1l to knock in a HygroR-T2A-PA tag cassette in-frame into the *Cep290* gene, following the first methionine codon (ATG) of the open reading frame (ORF) by non-homologous end joining (NHEJ). The *Cep290* allele was excised using the Cas9/sgRNA-CEP290-N complex. The donor vector containing the Hygro^R^-T2A-PA tag cassette flanked by two *tia1l* sequences was cleaved by the Cas9/sgRNA-tia1l complex. (B) Genotyping PCR analysis of wild-type and PA-CEP290 cells. PA-CEP290 cells harbor both wild-type and knock-in allele, indicating heterozygosity in this cell line. (C) The subcellular distribution of PA-CEP290 was confirmed by immunostaining. Green: PA-CEP290, Red: γ-Tubulin, and Blue: nucleus. Note that PA-CEP290 localizes to the centrosome, as marked by γ-tubulin (white arrowheads). (D) Colocalization of γ-tubulin, CP110 and PA-CEP290 (as shown in Fig 2B) were analyzed by Coloc 2 add-in of ImageJ/Fiji. Pearson’s colocalization coefficients for γ-tubulin and PA-CEP290, γ-tubulin and CP110, and CP110 and PA-CEP290 were compared and statistically analyzed via ANOVA with post-hoc Tukey tests. *: *p* < 0.01., N.S.: not significant. (E) Background noise of PA antibody staining was evaluated. Immunofluorescence staining was performed under the same conditions, and images were acquired with the same exposure time. White boxed regions were magnified in the lower right corners. Arrowheads indicate centrosomes. In wild-type cells, little PA antibody signal was observed, but in PA-CEP290 cells, PA antibody signal could be observed throughout the cell. (F) Fluorescence intensities originating from PA-tag in wild-type and PA-CEP290 cells, as shown in E, were compared. The data were obtained from three independent experiments and analyzed with Student’s *t*-test. *: *p *< 0.01. (G) Subcellular localization of PA-CEP290 was assessed by immunostaining under ciliated conditions. Cells were cultured until confluent followed by a medium change to fresh serum-free medium. After 24 hours of incubation, cells were fixed with 4% PFA and subjected to immunostaining with anti-Arl13b (green), anti-PA (red), anti-γ-tubulin (blue) and DAPI (grey). The scale bar represents 5 μm. The cilium in the yellow-boxed region is magnified and shown at the bottom. Note that PA-CEP290 was localized at the transition zone of a cilium. (H) The growth rate of wild type and PA-CEP290 cells were compared. Statistical analysis revealed no significant differences between the two cell types. Independent triplicate experiments were performed, with *n* = 4 at each time point. (I) Cell migration assay was performed in wild-type and CEP290 cells as described in Fig 2. No statistically significant differences were detected between the two cell types. Independent triplicate experiments were performed, with *n* = 30 at each time point.
(EPS)

S5 Fig Specificity of the interaction of PA-CEP290 with APCs. (A) To establish a stable cell line expressing EGFP with an N-terminal PA tag, mIMCD3 cells were transfected with pEGFP-N1-PA tag and selected with 400 ug/ml of G418. PA-EGFP expression was examined by immunofluorescent microscopy with anti-PA antibody. PA fluorescent signals were observed in EGFP-positive cells. Notably, the PA-tag alone did not localize specifically to the centrosome or the leading edge. (B) Immunoprecipitation assay of PA-EGFP and APC. APC did not co-immunoprecipitate by anti-PA antibody, excluding the possibility that APC were co-immunoprecipitated by nonspecific reaction of anti-PA antibodies.
(EPS)

S6 Fig Subcellular localization and protein level of APC in wild type and *Cep290* KO cells. (A) The subcellular localization of APC at the leading edge was assessed in wild type and *Cep290* KO cells. Cells were fixed with 4% PFA and immunostained with anti-APC antibody (green), anti-paxillin antibody (red) and DAPI (blue), respectively. The scale bar represents 10 μm. Regions of the leading edge enclosed by white square were magnified and are shown at the bottom right. (B) The fluorescence intensity of APC at the leading edge was quantified and statistically analyzed. Data were acquired from three independent experiments and analyzed by ImageJ/Fiji. Statistical significance among multiple groups were evaluated via ANOVA with post-hoc Tukey tests. *: *p* < 0.05. Note that fluorescence intensity of APC at the leading edge was significantly decreased in *Cep290* KO cells. (C) Protein expression levels in wild type and *Cep290* KO cells were assessed by Western blotting. Band densities of APC and p150^*Glued*^ were quantified using ImageJ/Fiji from three independent experiments. APC intensity values were normalized to those of p150^Glued^, and relative intensity values are shown below the Western blot images, with the wild-type value set to 1.0 for comparison. Statistical significance among multiple groups were analyzed via ANOVA with post-hoc Tukey tests. N.S.: not significant.
(EPS)

S7 Fig Line profile analysis of PA-CEP290 cells. Related to Fig 4J, we additionally analyzed line profiles of three different ROIs. Cells were immune-stained with anti-APC antibody (green), anti-PA antibody (red), and anti-α-tubulin antibody (gray), respectively.
(EPS)

S8 Fig Phenotype analysis of wild type and *Cep290* KO cells after nocodazole treatment. Related to Fig 5, we analyzed cell area (A) and cell number (B) of wild-type and *Cep290* KO cells after nocodazole treatment. The data were acquired from three independent experiments and analyzed by ImageJ/Fiji. Statistically significance among multiple groups were analyzed via ANOVA with post-hoc Tukey tests. *: *p* < 0.01.
(EPS)

S9 Fig Whole images of Western blot used in this study. The boxed areas in the gel images of (A), (B), and (C) were shown in Figs. 1B, 4A, and 4C, respectively.
(EPS)

S10 Fig Whole images of Western blot used in Supporting Information. The boxed areas in the gel images of (A) and (B) were shown in S3 Fig and S6 Fig, respectively.
(EPS)

S11 Movie Live imaging data of EB3-EGFP in the wild type cell. As shown in Fig. 1D, the 3D live imaging data of EB3-EGFP in wild type cells were orthogonally projected. Please note the velocity presented in Fig 1E was calculated in three-dimension space. Scale bar indicates 5 µm.
(AVI)

S12 Movie Live imaging data of EB3-EGFP in *Cep290* KO1 cell. As shown in Fig. 1D, 3D live imaging data of EB3-EGFP in *Cep290* KO1 cells were orthogonally projected. Please note the velocity presented in Figure 1E was calculated in three-dimension space. The scale bar indicates 5 µm.
(AVI)

S13 Movie Live imaging data of EB3-EGFP in *Cep290* KO2 cell. As shown in Fig. 1D, 3D live imaging data of EB3-EGFP in *Cep290* KO2 cells were orthogonally projected. Please note the velocity presented in Fig 1E was calculated in three-dimension space. Scale bar indicates 5 µm.
(AVI)
